# Characterizing Adolescent Social Media Experiences and Links to Momentary Affect

**DOI:** 10.1002/jad.12530

**Published:** 2025-06-05

**Authors:** Stefanie L. Sequeira, Kirsten M. P. McKone, Ella M. Diab, J. Graham Thomas, Jennifer Wolff, Jacqueline Nesi

**Affiliations:** ^1^ Department of Psychology University of Virginia Charlottesville Virginia USA; ^2^ Institute of Child Development University of Minnesota Minneapolis Minnesota USA; ^3^ Department of Psychiatry and Human Behavior Warren Alpert Medical School of Brown University Providence Rhode Island USA; ^4^ Department of Psychology Florida International University Miami Florida USA

## Abstract

**Introduction:**

Social media (SM) use is ubiquitous among adolescents, and questions remain regarding the impact of SM on youths’ emotional health. Understanding *what* adolescents are experiencing on SM is critical for answering these questions.

**Methods:**

The present study uses ecological momentary assessment (EMA) to characterize SM experiences (SMEs) over 15 days in 94 U.S. adolescents ages 12–15 years (53% female, 64% White). At each EMA observation, participants used a pre‐specified list to indicate which (if any) socially threatening and rewarding SMEs they had since the last observation. Online communication behaviors and valence‐ambiguous SMEs were also explored. In addition to characterizing the frequency of these SMEs, associations between SMEs and momentary positive and negative affect (PA/NA) were tested using multilevel modeling.

**Results:**

Adolescents more frequently reported rewarding (e.g., receiving supportive or encouraging comments) than threatening (e.g., getting bullied, blocked, or told mean comments) SMEs. Moreover, 41% of participants failed to endorse any threatening SMEs that were assessed over the 15‐day EMA period. While rewarding and threatening SMEs were associated with higher concurrent PA and NA, respectively, SMEs were not prospectively related to changes in within‐person affect from one survey to the next.

**Conclusions:**

Identifying what adolescents are experiencing when using SM is crucial for better understanding how SM might be impacting youths’ well‐being. Findings help characterize common SMEs among adolescents and suggest that even threatening SMEs may not drive short‐term changes in youths’ affective health.

## 1 Introduction

Use of social technology, including social media, is part of everyday life for most Americans, especially adolescents. As of 2023, 95% of U.S. adolescents report having access to a smartphone and around 50–60% of youth report using common social media platforms (i.e., Snapchat, TikTok, Instagram) every day, with nearly half of youth reporting that they use the internet “almost constantly” (Anderson et al. 2023). There is widespread interest in how social media impacts adolescents’ well‐being (Valkenburg et al. 2022b), though research tackling this question presents mixed and often complex findings with small effect sizes (Ivie et al. 2020; Meier and Reinecke 2021). Moreover, recent research highlights high heterogeneity, such that the impact of social media use on well‐being differs not only *between* youth (Beyens et al. 2020) but also *within* youth (Pouwels et al. 2024).

Critically, much of the existing research examining links between social media and well‐being operationalizes social media use as total time spent on social media. Increasingly, there have been calls to move away from focusing solely on total time on social media to prioritize studying *what* youth are experiencing on social media and *why* these experiences may be harmful or beneficial for emotional health (Valkenburg et al. 2022b; Vidal et al. 2020; Wirtz et al. 2021). For example, a growing body of research on youth suggests that engaging in negative social comparison when using social media is linked to poorer mental health (e.g., de Vries and Kühne 2015; Holland and Tiggemann 2016; Nesi and Prinstein 2015), while directly engaging with others (e.g., by posting, direct messaging) may be linked to better emotional well‐being (Ferguson et al. 2024; Keum et al. 2023). The present descriptive study builds on a growing area of research on adolescent social media experiences (SMEs) by using ecological momentary assessment (EMA) to characterize socially threatening and rewarding SMEs in a sample of early adolescents (ages 12–15 years) and test preliminary associations between these experiences and momentary positive affect (PA) and negative affect (NA).

### 1.1 Social Threat and Reward on Social Media

Most existing studies on SMEs in youth focus on youths’ cognitive responses to social media (e.g., tendency to engage in negative social comparison) (de Vries and Kühne 2015; Nesi and Prinstein 2015), emotional responses to social media (e.g., availability stress, feeling left out) (Dam et al. 2023; Giray et al. 2024; Mulvey et al. 2017), or behaviors on social media (e.g., making or viewing posts, liking or commenting, checking likes, direct messaging) (Colasante et al. 2024; Ferguson et al. 2024; Valkenburg et al. 2022a). In the present study, we focus primarily on assessing specific socially threatening and rewarding experiences that might *happen to youth* on social media (e.g., getting blocked, getting into a disagreement, being told something supportive). The identification of these specific SMEs is informed by recent theoretical models (Orben et al. 2024; Nesi et al. 2018a, 2018b) that discuss how the features of social media may create unique social challenges for youth online. For example, the asynchronicity, permanence, publicness, and availability associated with social media might make threatening online social experiences (e.g., bullying, harassment) more frequent, sustained, repetitive, and/or visible, and increase youths’ exposure to threatening content (e.g., violence) online. The visual nature and lack of interpersonal cues (e.g., vocal tone, physical touch, facial expressions) when communicating on social media may also create harsher victimization experiences and more opportunities for different types of victimization, while the quantifiability (e.g., number of views, likes, or comments) could reinforce perpetrators (Nesi et al. 2018b). Research has shown that cyberbullying on social media is consistently linked to higher depressive symptoms, self‐harm, and suicidal ideation in adolescents, particularly LGBTQ+ adolescents (Bonsaksen et al. 2023; Cole et al. 2016; Duarte et al. 2018; Hamm et al. 2015; Nesi et al. 2021; Sampasa‐Kanyinga and Hamilton 2015; Vidal et al. 2020). The availability, publicness, and quantifiability of social media may also contribute to youth more easily and frequently feeling left out or excluded (Orben et al. 2022). For example, youth might view photos or see the location of their friends interacting without them. Research has shown that such feelings of exclusion or fear‐of‐missing‐out (FOMO) are linked to poorer well‐being in youth (Dam et al. 2023; Mulvey et al. 2017).

These features of social media can also support rewarding SMEs. For example, the publicness of social media can enhance exposure to positive and inspiring content. Further, the immediacy and ubiquity of social media contribute to a greater potential for immediate social support and new opportunities for connecting with peers. In a qualitative study of adolescents hospitalized for suicidality, youth reported that social media provides them with social connection, social support, and content to boost their mood (Weinstein et al. 2021). We still know little about 1) how frequent these threatening (e.g., being bullied) or rewarding (e.g., seeing or being sent something funny) experiences might be occurring via social media for youth, or 2) the acute impact of these experiences on everyday affective well‐being. However, promising recent EMA research has found that when youth have more positive responses to SMEs than usual, they report higher PA (Boyd et al. 2024).

#### 1.1.1 Assessing SMEs With Ecological Momentary Assessment (EMA)

Though limited in number, some studies have assessed longitudinal associations between social media experiences/behaviors and adolescent well‐being. For example, a recent multi‐year study of youth ages 10–16 found no evidence for longitudinal associations between social media behaviors (i.e., frequency of posting, liking, and commenting) and symptoms of depression or anxiety (Steinsbekk et al. 2023). A second study of youth around age 14 found that higher depressive symptoms were associated with more negative responses to SMEs 1 year later while more positive responses to SMEs were associated with higher depressive symptoms 1 year later (Nesi et al. 2022). As research suggests that social media use, SMEs, and emotions fluctuate over short periods of time (Boyd et al. 2024; Ferguson et al. 2024; Hamilton et al. 2023), one question deserving greater attention is whether SMEs have more *proximal* impacts on youths’ emotional health.

EMA presents an ideal method for assessing the frequency of different SMEs and links to proximal changes in affect. Using EMA, participants can report on their SMEs multiple times per day (often for multiple days or weeks) while in their natural environments; relative to traditional questionnaire methods, this approach reduces recall bias, improves ecological validity, and allows for an assessment of how SMEs vary over time. EMA also allows for disaggregating within‐ and between‐person effects of SMEs on affect, which is critical as recent research suggests that the impacts of SMEs on affect may be stronger at the within‐person level than the between‐person level (Ferguson et al. 2024). Though most existing EMA studies on social media in adolescents have focused on assessing time spent on social media (e.g., Armstrong‐Carter et al. 2023; Dreier et al. 2023; Hamilton et al. 2020, 2023; Nereim et al. 2022; Taylor et al. 2024; Rodman et al. 2024), several recent EMA studies have integrated measures of SMEs (Boyd et al. 2024; Ferguson et al. 2024) to examine proximal associations between SMEs and well‐being. To date, these EMA studies have focused on behaviors on social media (e.g., posting, browsing, checking likes) (Colasante et al. 2024; Ferguson et al. 2024; Valkenburg et al. 2022a) or emotional responses to SMEs (e.g., feeling worried about missing out on things; feeling happy or excited because of a positive interaction; liking a positive interaction) (Boyd et al. 2024; Valkenburg et al. 2021). One limitation of these latter studies is that imbuing NA (e.g., worry) or PA (e.g., happy or excited) in the assessment of SMEs may bias associations found between these experiences and momentary affect. To address this limitation and extend prior EMA research beyond screen time and social media behaviors, the present study assesses more specific threatening and rewarding SMEs (e.g., getting blocked) and links these SMEs to fluctuations in PA and NA using EMA.

#### 1.1.2 Relevance to Early Adolescence

We focus this study on early adolescents ages 12–15. During the transition from childhood to early adolescence, youth experience substantial social, cognitive, and emotional changes, related in part to significant neurobiological maturation occurring with the onset of puberty and surges in pubertal hormones (Blakemore et al. 2010). As they enter middle and high school, youth show heightened sensitivity to social evaluation, which helps them navigate new, complex social environments to form new relationships and attain social status (Somerville 2013). Today, social media is a key component of these social environments for most youth. Indeed, many adolescents start opening their own social media accounts around ages 12–13 (Lauricella et al. 2016), with social media use generally increasing from ages 12 to 15 (Anderson et al. 2023). Given the strong social motivation and social goals that characterize this developmental period, along with ongoing changes in cognitive and emotional development, early adolescents may be particularly responsive to and impacted by threatening and rewarding social media experiences (Orben et al. 2022). Further, perceiving frequent threatening and rewarding experiences on social media could help explain increases in mental health concerns, including anxiety, depression, and body image concerns, seen in this age group (Merikangas et al. 2010; Westerberg‐Jacobson et al. 2012).

#### 1.1.3 Present Study

In the present study, largely descriptive in nature, we used a 2‐week EMA protocol to assess a subset of SMEs in youth ages 12–15 years, focusing on socially threatening and rewarding experiences that may be particularly salient for early adolescents given their heightened sensitivity to social cues and feedback (e.g., getting into a disagreement, getting bullied, receiving support from others). Consistent with other studies (e.g., Nesi et al. 2021), social media was defined as applications or websites that involve social interaction, such as Snapchat, TikTok, Instagram, and text messaging. Consistent with our analytic preregistration, our first goal was to characterize the frequency at which these SMEs are reported by adolescents, with considerations given to potential differences based on sex and age. Research suggests that relative to adolescent boys, adolescent girls use social media more frequently (Nesi et al. 2022) and are more sensitive to social evaluation in person and online (Choukas‐Bradley et al. 2022, 2023; Nesi et al. 2022). Younger adolescents may also have different experiences than middle adolescents, which may contribute to research showing that the link between social media use and psychological problems is stronger in younger adolescents (Orben et al. 2022; Tsitsika et al. 2014). Though we focus on social threat and reward, we also assess the frequency of youths’ social media use, online communication behaviors (e.g., posting), and other SMEs that may be more ambiguous in valence (i.e., not clearly threatening or rewarding, such as viewing posts about mental health) to achieve a more thorough picture of youths’ social media use and experiences.

The second preregistered goal of the present study was to test concurrent and prospective associations between SMEs and momentary PA and NA. We hypothesized that reporting more threatening SMEs would be associated with higher concurrent NA and prospective increases in NA, while reporting more rewarding SMEs would be associated with higher concurrent PA and prospective increases in PA (at both within‐ and between‐person levels). In exploratory analyses, we also examined concurrent associations between affect and 1) online communication (i.e., talking with others or posting online) and 2) valence‐ambiguous SMEs, such as being pulled into drama (i.e., becoming part of an ongoing interpersonal conflict), which could be either rewarding or threatening (or both), as it could enhance connection while causing stress (Marwick and Boyd 2014). Finally, we examined lagged associations between affect and social media use (i.e., affect at the previous timepoint predicting social media use) in an exploratory fashion to examine whether youth may be more likely to engage with social media depending on their affective state.

## 2 Methods

### 2.1 Participants

A total of 102 adolescent‐caregiver dyads were recruited from local primary care clinics and advertisements on Facebook and Instagram for a larger study of parenting practices and social media use. Inclusion criteria included: adolescent age between 12 and 15 years, English or Spanish fluency, parent and adolescent regular access to smartphones, and adolescent use of at least one social media platform at least once a day. Participants who completed at least 10% of EMA assessments were included in analyses. Of the 102 recruited adolescent participants, 10 did not complete any EMA assessments and two participants completed less than 10% of assessments, for a final analysis sample of 94 participants (*n* = 50 assigned female at birth). Participant demographics are described in Table 1. Adolescents included in analyses were 13.28 (SD = 1.11) years on average and the majority identified as cisgender (90.4%) and white (63.8%); 33% identified as Hispanic or Latinx. Although a non‐negligible proportion of our sample identified in a manner other than cisgender, the small group size precluded our ability to reliably examine group differences based on gender identity compared to sex assigned at birth. As a result, as noted below, we opted to include sex assigned at birth in analyses.

**Table 1:** Demographic characteristics of the final sample (*N* = 94).

	Mean (SD)
Age	13.28 (1.11)
	*N* (%)
Sex	
Female	50 (53.2%)
Male	44 (46.8%)
Income	
Less than $10,000	4 (4.3%)
$10,000–$25,999	11 (11.8%)
$26,000–$49,999	15 (16.1%)
$50,000–$74,999	12 (12.9%)
$75,000–$99,999	12 (12.9%)
$100,000–$149,999	22 (23.7%)
$150,000 or more	17 (18.3%)
Race/ethnicity	
American Indian or Alaska Native	3 (3.2%)
Asian	4 (4.3%)
Black	18 (19.1%)
Middle Eastern	1 (1.1%)
Native Hawaiian	1 (1.1%)
White	60 (63.8%)
Other	14 (14.9%)
Hispanic or Latine	31 (33.0%)

### 2.2 Procedures

Analyses for this study were pre‐registered following the completion of data collection. The study pre‐registration and code used for analyses can be found here: https://osf.io/745cx/?view_only=9b2f076e71e74668824ccbc85d84db18.

Prior to the initiation of study procedures, adolescent participants provided informed assent and their parent/caregiver provided informed consent. Adolescents completed a series of questionnaires at baseline. Pertinent to this study, questionnaires included a demographic survey including self‐reported age and sex assigned at birth. Caregiver procedures are not relevant to the current report and will not be discussed in further detail. As part of the EMA protocol, adolescents responded to prompts delivered to their personal smartphones 3–5 times per day for 15 consecutive days^1^. On weekdays, adolescents were prompted to complete 3 assessments per day, limited to after school hours. On “weekend” days (including holidays and school vacations) participants received 5 prompts/day. The timing of prompts was semi‐random, anchored at 4 pm, 7 pm, and bedtime (determined in collaboration with parents and adolescents) on weekdays, plus 11am and 1 pm on weekends. To introduce uncertainty, sampling times were drawn randomly from a normal distribution centered on each anchor time using a standard deviation of 30 min. Because participants started data collection on different days and only some participants completed data collection on holidays, the total number of observations differed across participants. For the final sample (n = 94), compliance rates were high, with an average compliance rate of 79.7%, and the number of surveys completed by each participant ranged from 6 to 59.

Participants self‐reported their affect at each EMA prompt. Additionally, at each EMA observation, participants responded to a question asking whether they used social media since the previous observation. For prompts in which the adolescent responded that they had used social media since the previous observation, they responded to 15 additional questions asking about various experiences adolescents might have on social media. These questions were coded into four variables reflecting rewarding experiences, threatening experiences, online communication behaviors, and valence‐ambiguous experiences (see “Measures”). Each question was on a dichotomous scale such that *no* indicated they did not have that experience and *yes* indicated they did have that experience since the last observation.

Adolescents and their caregivers were compensated $50 each for their time during the baseline assessment. Each dyad was also paid an additional amount based on the number of EMA surveys they completed, resulting in a maximum of $92 for the adolescent and $70 for the caregiver.

### 2.3 Measures

#### 2.3.1 Momentary Positive and Negative Affect

At each EMA observation, participants reported their current affect using items adapted from the Positive and Negative Affect Scale for Children (PANAS‐C; Laurent et al. 1999), with “happy” used as a measure of PA and the mean of “mad,” “sad,” “worried,” and “lonely” used as a measure of NA. Multilevel reliability analysis indicated that the measure of NA was adequately reliable at both the between (*ɑ* = 0.81, *ω* = 0.83) and within‐person levels (*ɑ* = 0.67, *ω* = 0.68).

#### 2.3.2 Social Media Experiences (SMEs)

EMA items of SMEs were developed based on a review of prior literature; several of these have been used in prior research (Nesi et al. 2019).

##### 2.3.2.1 Rewarding SMEs

Three items comprised the rewarding SME category: “People said supportive or encouraging things to me”, “Someone shared something funny or entertaining with me”, and “I saw something that made me feel happy or inspired”. As the third item was not interactive in nature, pre‐registered sensitivity tests were conducted removing the item from analyses (i.e., multilevel models) to ensure findings were robust. Based on our pre‐registered analytic approach (see below), rewarding SMEs were coded as 1 = experienced one or more rewarding SMEs at the given timepoint, and 0 = did not report any rewarding SMEs at the given timepoint.

##### 2.3.2.2 Threatening SMEs

Six items assessed threatening SMEs: “I got blocked, unfriended, or kicked out of a group chat, discussion board, or game”; “I got into a fight, argument, or disagreement”; “Someone spread rumors about me or shared something I thought was private (e.g., a photo or conversation) with others”; “I got bullied, harassed, or teased”; “Someone made negative comments or said mean things to me”; “I saw something that made me feel upset or uncomfortable”. Similar to the rewarding SME items, as the final item was not interactive in nature, pre‐registered sensitivity tests were conducted removing this item. Similar to the rewarding SMEs item, threatening SMEs were coded as 1 = experienced one or more threatening SMEs at the given timepoint, and 0 = did not report any threatening SMEs at the given timepoint.

##### 2.3.2.3 Online Communication Behaviors

Three items assessed online communication: “I posted something online”; “I talked directly (e.g., via messages) with a friend”; “I talked to someone I've never met in person (i.e., an online‐only friend)”. The online communication item was coded as 1 = engaged in one or more online communication behaviors at the given timepoint, and 0 = did not report any online communication at the given timepoint.

##### 2.3.2.4 Valence‐Ambiguous SMEs

Three items assessed other experiences on social media that could be positive or negative in valence: “I got pulled into drama”; “I saw other people posting about drugs or alcohol”; “I saw people posting about their mental health”. The valence‐ambiguous SMEs item was coded as 1 = experienced one or more valence‐ambiguous SME at the given timepoint, and 0 = did not report any valence‐ambiguous SME at the given timepoint.

### 2.4 Analytic Approach

#### 2.4.1 Preliminary Analyses

Consistent with our pre‐registration, descriptive statistics and frequency of endorsement within each SME category (i.e., rewarding, threatening, online communication, valence‐ambiguous) were calculated to identify how often adolescents reported multiple experiences within a given category at the same observation. Most EMA ratings were characterized by either zero or one SME in each category (Table 2). As a result, SME categories were modeled as binary variables where 0 = did not have an experience of this type in this moment, and 1 = had an experience of this type in this moment.

**Table 2:** Descriptive data on frequency of social media use and experiences.

Social media use frequency	Within‐person % of completed EMA calls with SM use endorsed, Mean (SD)		Sex diff?	Age diff?
Total Sample	59% (31%)		Y	N
Females	66% (29%)			
Males	50% (31%)			
Older (14–15 years)	62% (28%)			
Younger (12–13 years)	57% (33%)			

**Social Media Experiences (SMEs)**	**Within‐person % of completed EMA calls with SME endorsed, Mean (SD)**	**Participants endorsing SME at least once, *N* (%)**	**Sex diff?**	**Age diff?**
*Threatening SMEs*				
ANY	10% (15%)	55 (59%)	N	N
I saw something that made me feel upset or uncomfortable	6.1% (12%)	37 (39%)		
I got into a fight, argument, or disagreement	2.8% (6.3%)	30 (32%)		
I got bullied, harassed, or teased	0.5% (2.3%)	7 (7%)		
I got blocked, unfriended, or kicked out of a group chat, discussion board, or game	1.2% (4.0%)	15 (16%)		
Someone made negative comments or said mean things to me	1.3% (3.4%)	17 (18%)		
Someone spread rumors about me or shared something I thought was private (e.g., a photo or conversation) with others	0.9% (3.1%)	11 (12%)		
*Rewarding SMEs*				
ANY	43% (31%)	85 (90%)	N	Y
I saw something that made me feel happy or inspired	32% (30%)	75 (80%)		
Someone shared something funny or entertaining with me	22% (23%)	73 (78%)		
People said supportive or encouraging things to me	9.1% (14%)	54 (57%)		
*Online Communication Behaviors*				
ANY	51% (31%)	84 (89%)	Y	Y
I talked directly (e.g., via messages) with a friend	43% (31%)	82 (87%)		
I posted something online	13% (19%)	56 (60%)		
I talked to someone I've never met in person (i.e., an online‐only friend)	8.1% (18%)	39 (42%)		
*Valence‐Ambiguous SMEs*				
ANY	15% (21%)	59 (63%)	N	N
I saw people posting about their mental health	12% (20%)	46 (49%)		
I got pulled into drama	1.4% (3.7%)	16 (17%)		
I saw other people posting about drugs or alcohol	4.7% (11.2%)	25 (27%)		

*Note:* “Sex diff” or “Age diff” columns indicate whether or not significant sex or age differences emerged (Y = yes, N = no); more information on differences that emerged can be found in the main text.

Abbreviations: SM = social media, SME = social media experience.

#### 2.4.2 Primary Analyses

We conducted our analyses in stages, consistent with our hypotheses and pre‐registration.

First, we conducted descriptive analyses to characterize the frequency of social media use and all SMEs (i.e., rewarding, threatening, online communication, valence‐ambiguous), as well as examine differences in frequency of social media use and experiences by sex and age. To test age differences, participants were assigned to one of two groups: younger adolescents (ages 12–13; *N* = 55) and older adolescents (ages 14–15; *N* = 39). Younger adolescents (primarily in middle school) may spend less time on social media (Anderson et al. 2023) and have different social experiences relative to older adolescents (primarily in high school). Dichotomizing age was done only for descriptive purposes to improve interpretability of the descriptive statistics; in multilevel models described below, age was entered as a continuous variable. To empirically test differences between groups in a descriptive fashion, t‐tests were conducted using person‐means to address the repeated‐measures nature of the data.

In our second analytic stage, concurrent associations between SMEs and affect were tested in a two‐step approach. In the initial step, bivariate multilevel correlations were conducted in Mplus (Muthén and Muthén 2017) with observations nested within individuals. Subsequently, multivariate tests of significant concurrent bivariate associations were conducted to test the robustness of bivariate associations to the inclusion of covariates (age, sex, time since report). Additionally, we conducted two exploratory sensitivity analyses of concurrent associations that were not pre‐registered, namely including lagged affect as a predictor of concurrent affect in each of our tests for robustness, considering autoregressive effects of affect may confound the true effect of SME on concurrent affect. An example model equation for concurrent associations with all tested covariates (including the exploratory autoregressive affect) with threatening SMEs as the predictor of interest is included below. The primary parameters of interest are β_3_, which represents the within‐person effect of threatening SMEs on NA at the current timepoint, and y_30_, which indicates the between‐person effect of threatening SMEs on current NA, and β_2,_ which indicates the autoregressive effect of NA.

#### 2.4.3 Concurrent Multilevel Model Example

Level 1: NA_*tj*_ = β_0*dj*_ + β_1_Time_*t*_ + β_2_NA_(t‐1)j_ + β_3_ Threatening Social Media Experience(s)_*tj*_ + e_*tj*_

Level 2: β_0*j*_ = y_00_ + y_01_AgeGMC_*j*_ + y_02_Sex_*j*_ + y_03_ Threatening Social Media Experience(s)PM_*j*_ + u_0*j*_

β_0*dj*_ = β_0*j* +_ u_0*dj*_

β_1_ = y_10_ + u_1*dj*_

β_2_ = y_20_

β_3_ = y_30_

Third, we examined prospective associations between threatening and rewarding SMEs and affect at the subsequent timepoint, adjusting for the same covariates. The primary parameters of interest are β_3_, which represents the within‐person effect of threatening SMEs at the previous timepoint on NA at the current timepoint, and y_03_, which indicates the between‐person effect of threatening SMEs on current NA, and β_1,_ which indicates the autoregressive effect of NA.

#### 2.4.4 Prospective Multilevel Model Example

Level 1: NA_*tj*_ = β_0*dj*_ + β_1_Time_*t*_ + β_2_NA_(t‐1)j_ + β_3_ Threatening Social Media Experience(s)_(*t*‐1)*j*_ + e_*tj*_

Level 2: β_0*j*_ = y_00_ + y_01_AgeGMC_*j*_ + y_02_Sex_*j*_ + y_03_ Threatening Social Media Experience(s)PM_*j*_ + u_0*j*_

β_0*dj*_ = β_0*j* +_ u_0*dj*_

β_1_ = y_10_ + u_1*dj*_

β_2_ = y_20_

β_3_ = y_30_

Finally, a pre‐registered set of exploratory analyses examined potential bidirectional effects, by examining associations between lagged affect and concurrent use of SM (i.e., testing whether affect at the previous timepoint predicts use of SM at the current timepoint). A sample model equation is included below. The primary parameters of interest are β_2_ and y_03_, which represent the within‐ and between‐person effects of lagged NA on current SM use, respectively.

#### 2.4.5 Affect Predicting SM Use Multilevel Model Example

Level 1: SM Use_*tj*_ = β_0*j*_ + β_1*j*_Time_*t*_ + β_2_NA PMC_(*t*‐1)*j*_ + e_*tj*_

Level 2: β_0j_ = y_00_ + y_01_AgeGMC_*j*_ + y_02_Sex_*j*_ + y_03_NA PM_*j*_ + u_0*j*_

β_0*dj*_ = β_0*j* +_ u_0*dj*_

β_1j_ = y_10_ + u_1dj_

β_2j_ = y_20_

β_3j_ = y_30_

All multivariate analyses were conducted using multilevel modeling in R (lme4 package, Bates et al. 2015; R version 4.2.1; R Core Team 2022) with unstructured variance‐covariance structure and allowing random intercepts and slopes to covary, to accommodate the dependency of repeated observations clustered within individuals. We additionally tested a second level of dependence, days within persons (i.e., random intercept and slope of day), that was erroneously left out of our pre‐registration. Hence, our models (with exceptions noted below) included a random intercept of person, as well as random intercept and slope of days within person. We did not otherwise model any random effects. Adding a random effect of day improved model fit for means‐only models testing associations between SMEs and affect; however, several models did not converge. We suspect this resulted from model overfitting. In such cases, we removed the random effect of day. Instead of including a fixed effect of day, we included a fixed effect of continuous study time (measured to the second), to facilitate interpretation of lagged effects. Additionally, we pre‐registered a plan to conduct a sensitivity analysis to evaluate whether results changed depending on whether it was a weekday versus weekend day, considering systematic differences in social media use.

Predictors were disaggregated into between‐ and within‐person effects by including the person‐mean of each type of social media experience as the between‐person effect. The original dichotomous variable was included to capture the within‐person effect. For all lagged analyses, the final timepoint of each day was excluded from analyses to avoid biasing estimates. Sensitivity analyses also included an adjustment for day type (weekend/holiday vs. weekday). Two models with directional hypotheses were tested, in addition to four exploratory models. Consistent with our pre‐registration, we interpreted effects to be meaningful at *p* < 0.05 for directional hypotheses and if the 95% confidence interval did not include zero for nondirectional/exploratory analyses.

### 2.5 Missing Data

As participants varied in the day they started EMA, there were differences in the number of total weekday versus weekend prompts, resulting in variation in total number of prompts participants received. Participants were prompted between 44 and 72 observations each, resulting in a total of 5210 possible prompts. One participant completed several extra days of data collection due to a technical error. Visual inspection indicated these data were valid; as this variability did not differ considerably from the variability inherent to the designed data collection method, this participant and their complete data were retained. Of the possible 5210 prompts, 96 calls nested in two participants were dropped because of response rates less than 10% and 1428 of the remaining calls went unanswered. The final analysis sample for multilevel models comprised 3660 observations contributed by 94 participants. As social media use and experiences differ between weekdays and weekend days (You et al. 2023), a binary variable indicating type of day (weekday vs. weekend) was included in a sensitivity analysis. We examined primary study variables (age, sex, SMEs, average PA/NA) to see if any were associated with EMA variables; none were significant predictors of missingness.

## 3 Results

### 3.1 Descriptive Data

#### 3.1.1 Social Media Use

Descriptive data on frequency of social media use can be found in Table 1. On average, participants reported that they used social media in the time since the last observation on 59% of completed surveys on average, with substantial variability in the percentage of occasions on which participants reported social media use (SD = 31%; median = 56%; mode = 100%; range = 2.3–100%). Females reported that they had used social media on a higher proportion of completed calls relative to males (*t*(92) = −2.59, *p* = 0.011, Cohen's *d* = −0.54). Social media use did not differ between older (14–15) and younger (12–13) participants (*p* = 0.44).

#### 3.1.2 SMEs

Descriptive data can be found in Table 2. Participants reported rewarding SMEs more frequently than threatening SMEs (paired samples *t*(93) = 11.23, *p* < 0.001, Cohen's *d* = 1.36). About 41% of participants (*N* = 39) denied all six threatening SMEs over the entire assessment period, while under 10% (*N* = 9) denied all three rewarding SMEs. Older participants reported rewarding SMEs more frequently on average (*M* = 51% of observations, SD = 25%) than younger participants (*M* = 38% of observations, SD = 34%; *t*(92) = −2.02, *p* = 0.046, Cohen's *d* = −0.43); there were no differences by sex *(p* = 0.33). No differences in frequency of reported threatening SMEs were seen by age (*p* = 0.26) or sex (*p* = 0.95).

Most participants (around 89%) engaged in online communication (i.e., posting online or talking with a friend) at least once during the EMA protocol; most often, this was talking directly with a friend. Older participants engaged in online communication more frequently on average (*M* = 62% of observations, SD = 28%) than younger participants (*M* = 44% of observations, SD = 31%; *t*(92) = −2.82, *p* = 0.006, Cohen's *d* = −0.60). Females engaged in online communication more frequently on average (*M* = 60% of observations, SD = 30%) than males (*M* = 42% of observations, SD = 29%; *t*(92) = −2.84, *p* = 0.005, Cohen's *d* = −0.59). Almost two‐thirds of participants reported at least one valence‐ambiguous SME; this was often seeing someone posting about mental health. There were no differences in frequency of reporting other SMEs by age (*p* = 0.69) or sex (*p* = 0.18).

### 3.2 Multilevel Correlations

Multilevel correlations between study variables are located in Table 3.

**Table 3:** Bivariate correlations between variables.

Within‐person associations above the diagonal; Between‐person associations below the diagonal						
Variable	1	2	3	4	5	6
1. Rewarding SME	—	0.04 [−0.08, 0.16]	**0.38 [0.30, 0.47]**	**0.16 [0.05 0.27]**	**0.13 [0.07, 0.20**]	−0.01 [−0.08, 0.06]
2. Threatening SME	**0.48 [0.25, 0.66]**	—	**0.15 [0.03, 0.27]**	**0.22 [0.08, 0.35]**	‐0.07 [−0.15, 0.01]	**0.27 [0.20, 0.35]**
3. Online Communication	**0.41 [0.20, 0.59]**	**0.34 [0.08, 0.56]**	—	0.03 [−0.09, 0.16]	**0.08 [0.01, 0.14]**	**0.10 [0.03, 0.18]**
4. Valence‐ambiguous SME	**0.51 [0.30, 0.67]**	**0.73 [0.56, 0.85]**	**0.44 [0.21, 0.63**]	—	−0.07 [−0.15, 0.02]	0.07 [‐0.02, 0.16]
5. PA	**0.25 [0.03, 0.45]**	‐0.25 [−0.48, 0.01]	0.12 [−0.12, 0.33]	‐0.23 [‐0.45, 0.01]	—	**−0.15 [−0.19, −0.12]**
6. NA	0.08 [−0.14, 0.30]	**0.27 [0.02, 0.47]**	0.09 [−0.14, 0.30]	0.14 [‐0.11, 0.36]	−0.07 [−0.26, 0.14]	—

*Note: N* = 3,660 observations. Values in brackets are the 95% confidence intervals and values in bold are those for which the confidence interval does not contain zero.

#### 3.2.1 Within‐Person

At the within‐person level, PA was inversely associated with NA. Further, reporting a rewarding SME at a given observation was associated with engaging in online communication and reporting a valence‐ambiguous SME at the same observation. Consistent with hypotheses, reporting a rewarding SME was associated with higher levels of concurrent PA. Reporting a threatening SME at a given time point was also associated with engaging in online communication and reporting a valence‐ambiguous SME at the same timepoint. Consistent with hypotheses, reporting a threatening SME was also associated with higher levels of concurrent NA. Exploratory analyses indicated that engaging in online communication was associated with higher levels of PA *and* higher levels of NA at the within‐person level.

#### 3.2.2 Between‐Person

At the between‐person level, having more rewarding SMEs was associated with having more threatening and valence‐ambiguous SMEs, as well as engaging in more online communication. Additionally, consistent with hypotheses, having more rewarding SMEs on average was associated with higher levels of PA on average. Reporting more threatening SMEs on average was also associated with reporting more online communication and valence‐ambiguous SMEs on average. Consistent with hypotheses, reporting more threatening SMEs was associated with higher levels of NA on average. Finally, reporting more online communication on average was associated with reporting more valence‐ambiguous SMEs on average. However, in contrast to the within‐person level, there were no between‐person associations between average levels of online communication and average levels of positive or NA.

### 3.3 Multilevel Models

#### 3.3.1 Robustness Tests of Concurrent Bivariate Associations

As noted in the analytic approach, multilevel models were run to test the robustness of concurrent bivariate associations between SMEs and affect in the context of covariates (i.e., age, sex) and the autoregressive effect of affect (exploratory analysis, not pre‐registered). Tests of concurrent associations are included in Table 4. When age, sex, and lagged PA were included in the model, associations between rewarding SMEs and concurrent PA remained significant at the within‐person level, but not at the between‐person level. Similarly, threatening SMEs remained associated with concurrent NA at the within‐person level, but not at the between‐person level when age, sex, and lagged NA were included in the model. Online communication, by contrast, was no longer associated with NA at the within‐person level when covariates were included in the model; however, online communication remained significantly positively associated with PA at the within‐person level, above and beyond the effects of covariates. Sensitivity tests adding weekend day and examining the rewarding and threatening experiences variables without the non‐social items did not change the results.

**Table 4:** Concurrent associations between social media experiences and affect adjusting for covariates.

	Negative Affect						Positive Affect					
Predictors	*Est*.	*CI*	*p*	*Est*.	*CI*	*p*	*Est*.	*CI*	*p*	*Est*.	*CI*	*p*
Intercept	0.74	0.63–0.85	**< 0.001**	0.72	0.60–0.84	**< 0.001**	1.98	1.71–2.26	**< 0.001**	2.00	1.72–2.28	**< 0.001**
Lagged Affect	0.36	0.31–0.42	**< 0.001**	0.39	0.33–0.44	**< 0.001**	0.35	0.30–0.40	**< 0.001**	0.35	0.30–0.40	**< 0.001**
Age	0.01	−0.04–0.06	0.725	0.03	−0.03–0.09	0.308	−0.09	−0.21–0.04	0.162	−0.09	−0.22–0.03	0.136
Sex	0.12	0.00–0.24	**0.046**	0.12	−0.01–0.25	0.071	−0.00	−0.28–0.27	0.976	−0.04	−0.32–0.24	0.786
Time	0.01	0.00–0.02	**0.031**	0.01	0.00–0.02	**0.025**	−0.02	−0.04–−0.01	**< 0.001**	−0.02	−0.04–−0.01	**< 0.001**
Avg. Threatening SME	0.29	−0.11–0.70	0.149									
Mom. Threatening SME	0.28	0.20–0.36	**< 0.001**									
Avg. Rewarding SME							0.25	−0.21–0.72	0.285			
Mom. Rewarding SME							0.18	0.06–0.31	**0.005**			
Avg. Communication SME				−0.05	−0.28–0.18	0.648				0.31	−0.19–0.82	0.225
Mom. Communication SME				0.02	−0.04–0.07	0.518				0.14	0.02–0.27	**0.025**
Random Effects												
σ^2^	0.14			0.14			0.74			0.74		
τ_00_	0.04 _Individual_			0.05 _Individual_			0.33 _Individual_			0.25 _Individual_		
τ_11_	0.00 _Individual. Day_			0.00 _Individual. Day_						0.00 _Individual. Day_		
ρ_01_	0.29 _Individual_			0.26 _Individual_						0.52 _Individual_		
ICC	0.23			0.24			0.30			0.25		
N	89 _Individual_			89 _Individual_			89 _Individual_			89 _Individual_		
Observations	1207			1207			1207			1207		
Marginal *R* ^2^/Conditional *R* ^2^	0.320/0.477			0.257/0.437			0.204/0.447			0.204/0.404		

*Note:* Bold values are statistically significant (*p* < 0.05).

Abbreviations: *Avg. = Average, CI = Confidence Interval, Est. = Estimate, Mom. = Momentary*, *SME = Social Media Experience.*

#### 3.3.2 Prospective Associations Between SMEs and Affect

In analyses examining rewarding and threatening SMEs at the previous timepoint predicting affect at the current timepoint, having a threatening SME at the previous timepoint was not associated with NA at the current timepoint (i.e., no within‐person association; Table 5). However, there was an effect at the between‐person level, such that participants who reported more threatening SMEs on average also reported higher levels of NA at the current timepoint, adjusting for within‐person effects of SMEs and NA. There were no significant associations between threatening SMEs and PA, or between rewarding SMEs and PA or NA.

**Table 5:** Prospective associations between social media experiences and affect.

	Negative affect			Positive affect		
Predictor	*Est*.	*CI*	*p*	*Est*.	*CI*	*p*
Intercept	0.64	0.52–0.76	**< 0.001**	2.04	1.76–2.32	**< 0.001**
Lagged Affect	0.49	0.44–0.55	**< 0.001**	0.01	−0.13–0.15	0.929
Age	−0.02	−0.07–0.03	0.481	−0.08	−0.19–0.04	0.217
Sex	0.06	−0.06–0.18	0.305	−0.02	−0.28–0.24	0.902
Time	0.01	0.00–0.01	**0.004**	−0.02	−0.04–‐0.01	**0.004**
Average Threatening SME	0.41	0.01–0.82	**0.045**			
Lagged Momentary Threatening SME	−0.04	−0.13–0.05	0.427			
Average Rewarding SME				0.35	0.29–0.41	**< 0.001**
Lagged Momentary Rewarding SME				0.46	0.01–0.91	**0.045**
Lagged PA						
Random Effects						
σ^2^	0.17			0.82		
τ_00_	0.06 _Individual_			0.23 _Individual_		
τ_11_				0.00 _Individual. Day_		
ρ_01_				0.06 _Individual_		
ICC	0.25			0.22		
N	91 _Individual_			91 _Individual_		
Observations	1119			1119		
Marginal *R* ^2^/Conditional *R* ^2^	0.309/0.480			0.204/0.379		

*Note:* Bold values are statistically significant (*p* < 0.05).

Abbreviations: *CI = Confidence Interval, Est. = Estimate, SME = Social Media Experience*.

#### 3.3.3 Lagged Affect Predicting Concurrent Social Media Use

Neither within−person NA nor PA from the previous timepoint was associated with social media use at the current timepoint. Average levels of affect were also not associated with social media use (Table 6).

**Table 6:** Prospective associations between affect and social media use.

	Social media use					
Predictor	*OR*	*CI*	*p*	*OR*	*CI*	*p*
Intercept	0.90	0.21–3.91	0.887	1.43	0.26–7.78	0.682
Age	1.12	0.75–1.68	0.579	1.13	0.75–1.69	0.568
Sex	2.84	1.12–7.20	**0.028**	2.91	1.15–7.35	**0.024**
Time	0.64	0.52–0.80	**< 0.001**	0.65	0.53–0.80	**< 0.001**
Weekend Day	0.80	0.64–1.01	0.064	0.80	0.63–1.01	0.058
Average NA	1.35	0.51–3.57	0.546			
Within‐person Lagged NA	1.13	0.84–1.52	0.406			
Average PA				0.98	0.59–1.64	0.944
Within‐person Lagged PA				1.02	0.90–1.15	0.764
Random Effects						
σ^2^	3.29			3.29		
τ_00_	4.17 _Individual_			4.14 _Individual_		
τ_11_	0.02 _Individual. Day_			0.03 _Individual. Day_		
ρ_01_	−0.26 _Individual_			−0.24 _Individual_		
ICC	0.56			0.56		
N	91 _Individual_			91 _Individual_		
Observations	2119			2119		
Marginal *R* ^2^/Conditional *R* ^2^	0.068/0.589			0.064/0.586		

*Note:* Bold values are statistically significant (*p* < 0.05).

Abbreviations: *CI = Confidence interval, NA = Negative affect, OR = Odds ratio, PA = Positive affect*.

### 3.4 Compliance Threshold Sensitivity Analysis

Upon request from a reviewer, we conducted a sensitivity analysis (not pre‐registered) using a more conservative compliance threshold of at least 20% compliance, which resulted in dropping *n* = 6 participants. Several bivariate correlations at the between‐person level were slightly altered in their magnitude and significance. Rewarding SMEs were still significantly positively associated with PA at the between‐person level; however, the 95% confidence interval included zero (*r* = 0.23, *p* = 0.026, 95% CI[−0.002, 0.44]). Threatening SMEs became significantly negatively associated with PA at the between person level, (*r* = −0.29, *p* = 0.013, 95% CI [−0.51, −0.03]), and valence‐ambiguous SMEs became significantly negatively associated with PA at the between‐person level (*r* = −0.27, *p* = 0.022, 95% CI[−0.48, −0.10]). No other bivariate correlations changed from the results presented in Table 3. None of the multilevel model results changed except for the significant effect of sex in the model testing prospective associations between positive affect and social media use (Table 6), which became nonsignificant at *p* = 0.052.

## 4 Discussion

Identifying what youth are experiencing on social media and how these experiences are linked to their daily affect can inform understanding of how social media may be impacting youths’ mental health. The present study measured fine‐grained and time‐varying SMEs via EMA, with the goal of better characterizing SMEs and examining preliminary effects of these experiences on daily PA and NA in a community sample of 12‐ to 15‐year‐olds. In characterizing SMEs, results suggest that youth reported rewarding SMEs at a higher frequency than threatening SMEs. Moreover, 41% of participants in this study did not endorse any threatening SMEs during the EMA period. This is particularly striking considering that this study assessed twice as many threatening SMEs as rewarding SMEs. However, many of the threatening experiences assessed may be considered particularly severe (e.g., being bullied or blocked); youth may be experiencing other, less acute or severe threatening experiences that were not assessed (in addition to rewarding experiences not assessed). For example, emerging research suggests that highly visual social media platforms (e.g., Instagram, Snapchat, TikTok) depicting edited images of youth and celebrities contribute to worse mental health in youth through increases in body image concerns (Choukas‐Bradley et al. 2023). Additionally, engaging in negative social comparison while using social media has been linked to increased depressive symptoms in adolescents, especially for girls and youth low in popularity or self‐esteem (Nesi and Prinstein 2015). Experiencing fear of missing out, or “FOMO”, has also been linked to changes in emotional health in adolescents (Einstein et al. 2023), and research suggests that experiencing digital stress (e.g., stress related to the pressure of responding to others or excessive notifications) impacts emotional health (Nick et al. 2022). Though youth may have considered these experiences when endorsing “I saw something that made me feel upset or uncomfortable,” it is important for future work to more explicitly assess experiences of social comparison, FOMO, and digital stress in assessments of threatening social media experiences.

Unsurprisingly, there was high variability in how often youth reported a rewarding or threatening SME. Relative to males, females endorsed using social media more often during the EMA assessment and reported more online communication, though no sex differences in reporting of rewarding or threatening SMEs were found. Younger adolescents (ages 12–13 years) did not differ from older adolescents (ages 14–15 years) in how frequently they reported threatening SMEs. However, relative to younger adolescents, older adolescents reported rewarding SMEs more frequently and engaged in online communication more often. Younger adolescents use most social media platforms at lower rates than older adolescents (Anderson et al. 2023) and may thus have fewer social connections on social media, thus fewer people to engage with. Findings also showed that at the between‐person level, youth who reported more rewarding SMEs also engaged in online communication more often; greater communication may create more opportunities for positive SMEs.

In addition to the descriptive goals of this study, we aimed to link socially threatening and rewarding SMEs to momentary PA and NA. As hypothesized, at the within‐person level, reporting at least one rewarding SME was associated with higher concurrent PA and reporting at least one threatening SME was associated with higher concurrent NA. Additionally, youth who reported more positive SMEs on average also reported higher PA on average and youth who reported more threatening SMEs on average also reported higher NA on average; of note, these between‐person effects did not survive when age, sex, and lagged affect were adjusted for, which could suggest that at the between‐person level, individual affective stability is a more robust predictor of concurrent affect than SMEs, and may shape how adolescents experience interactions on SM as rewarding or threatening. These findings are interesting to consider in relation to recent work by Ferguson et al. (2024), which failed to find any significant associations between social media behaviors (e.g., posting, checking likes) and NA at either within‐ or between‐ person levels, though several associations between these behaviors and PA were found. Together, findings may suggest that examining social media experiences, in addition to behaviors, is critical for understanding the emotional impacts of social media. Present findings also differ in interesting ways from recent work by Boyd et al. (2024), who showed that youth reported higher momentary PA and lower momentary NA when they reported more than their average positive SMEs, and higher momentary NA and lower momentary PA when they reported more than their average negative SMEs. In the present study, threatening SMEs were not associated with PA, nor were rewarding SMEs associated with NA. Differences in the assessment of SMEs between Boyd et al. (2024) and the present study may help account for these conflicting findings, as the SMEs assessed by Boyd et al. (2024) included an emotional component (e.g., “Thinking about the last time you used social media, how much did you feel sad or hurt because of a negative interaction (e.g., comment, post, DM) with someone else?”). Future work should attend closely not only to which SMEs are being assessed but also how they are being assessed.

In the present study, neither rewarding nor threatening SMEs were associated with *prospective* changes in affect from one timepoint to the next. One possibility is that rewarding and threatening SMEs only temporarily influence affect (i.e., concurrently). Another possibility is that youth with higher momentary NA hold a cognitive bias and are more likely to report threatening SMEs, while youth with higher momentary PA are more likely to report rewarding SMEs. We were unable to employ methods in this study that would allow us to speak more confidently to causality, such as cross‐lagged panel models, because SMEs were only reported on when youth used social media; thus, the spacing between measurements of affect and SMEs was uneven within and between participants.

Youth in this sample often messaged with friends on social media. Interestingly, exploratory analyses showed that online communication (i.e., talking with others, posting online) was associated concurrently with *both* higher NA and higher PA, though only associations with PA survived tests of robustness. Prior theory suggests that factors like the content of the communication influence whether communication will lead to positive or negative well‐being (Yang et al.^2021^). Moreover, interacting directly (e.g., messaging) with others could enhance social support and lead to better emotional health (Coyne et al. 2023), though engaging in difficult discussions or co‐rumination with others could lead to increased negative affect. Youths’ pre‐existing emotional state may also affect the impacts of messaging with others or posting on social media on affect. For example, youth who are feeling bad may choose to engage on social media to try to feel better by connecting with others, though some may engage in ineffective social emotion regulation strategies (e.g., co‐rumination) that maintain or exacerbate their negative mood. Future research in this area might explore what types of social media engagement are the most helpful for adolescents, and how we might harness this information to support positive youth outcomes.

The present study brings us closer to understanding what younger adolescents might be experiencing on social media. Strengths of this study include the focus on specific social media *experiences* and longitudinal assessment of affect and SMEs using EMA. However, this study is not without limitations. First, the sample size is relatively small at 94 participants, likely limiting our ability to detect small between‐person effects and limiting generalizations that can be made about SMEs in the general population of adolescents. However, these findings can be used to inform future research on SMEs in larger samples and youth diverse in geographic location, gender identity, and ethno‐racial identity. Second, because SMEs were measured only when youth used social media, our analyses cannot speak to causal links between SMEs and affect. Third, adolescents could only endorse a limited number of SMEs provided in a pre‐specified list, and youth may have had other meaningful SMEs not assessed; thus, we view these descriptive findings not as a definitive characterization of what youth are experiencing online, but as preliminary insight into the types of experiences youth ages 12‐15 are reporting, which can be further explored in future work. Finally, the affect variables used here were operationalized differently, with PA measured by a single item (happy) and NA measured as a composite of four negative emotions. Single‐item measures are often less reliable; hence, the findings on PA presented here may be less robust, as suggested by the slight changes in significance to the between‐person correlations between SMEs and PA when we applied a more conservative compliance threshold. Qualitative research and use of focus groups may also help us better understand the rewarding and threatening experiences adolescents are having on social media to improve future research, as well as the most effective (and least burdensome) ways to assess their emotional response to those experiences. Indeed, recent qualitative research has shed light on the risks and benefits adolescents associate with social media (Biernesser et al. 2022; de Felice et al. 2022; van der Wal et al. 2024).

Additionally, the grouping of SMEs into different categories was guided by prior theory and research but is not perfect, and there are likely other possible groupings. For example, in their Multidimensional Model of Social Media Use, Yang et al. (2021) distinguish between directed communication (e.g., messaging someone) and untargeted broadcasting (posting online). The authors propose that directed communication may be associated with better well‐being through perceived social support, while untargeted broadcasting can be associated with either positive or negative well‐being. In the present study, we pre‐registered our approach to examine all forms of online communication together; we decided not to separate different types of communication due to low rates of endorsement of communicating with an online‐only friend or posting online. However, future research may consider separating these constructs in larger datasets. Similarly, our exploratory valence‐ambiguous SME category was primarily included for descriptive purposes, and we chose not to examine how individual valence‐ambiguous experiences are associated with affect due to low rates of endorsement of each experience.

The SME items used in the study were also general and not culturally specific; youth with different cultural backgrounds and/or social identities may have unique experiences on social media that impact their well‐being. For example, some youth may experience higher exposure to violence, including racism or transphobia, on social media, which could contribute to high rates of mental health concerns in youth of color and LGBTQ+ youth. The present study did not assess time spent on social media; thus, it is not known whether associations between time on social media and affect would compare to associations between SMEs and affect. These variables (i.e., time on social media and SMEs) may provide complementary information and may be related, such that youth who use social media for more time may perceive more threatening and rewarding SMEs. Alternatively, youth who perceive more threatening SMEs may spend less time on social media, while youth who perceive more rewarding SMEs may spend more time on social media. Integrating measures of SMEs with other measures of social media use (e.g., time spent on social media, social media apps used) may further advance research in this area. Moreover, there may be other individual differences (e.g., internalizing symptoms, temperament, rejection sensitivity, parenting‐related factors, peer attachment) that might buffer or exacerbate the influence of social media experiences on well‐being (Valkenburg and Peter 2013). Future research may examine for whom and when certain social media experiences are helpful or harmful. Present findings provide a foundation for this future work to build upon.

## Ethics Statement

The study was approved by the Lifespan Institutional Review Board (IRB).

## Conflicts of Interest

The authors declare no conflicts of interest.

## Data Availability Statement

Code used for analyses can be found here: https://osf.io/745cx/?view_only=9b2f076e71e74668824ccbc85d84db18. Deidentified study data are available from the corresponding author by request.

## Endnotes

1 In the preregistration for this analysis, this was accidentally reported as 14 consecutive days.
